# Japanese Teeth

**Published:** 1885-05

**Authors:** 


### Japanese Teeth.

Before the Odontological Society of Great Britain (Dec. 1, 1884), Dr. St. George Elliott exhibited and presented to the museum some very curious artificial dentures of Japanese manufacture. These people had derived most of their technical and scientific knowledge from the Chinese, but, in this matter, they were far in advance of their teachers; for, whilst the latter could only carve a row of incisors and fasten them to the teeth on either side, the Japanese could make thoroughly serviceable dentures, and had been acquainted with the method of fixing them by suction for about two hundred years. The teeth were mounted on hard wood, those in front being made from quartz pebbles carefully ground down, but the process of mastication was performed by copper nails which occupied the place of the molars. One of these dentures had been in use for fifteen years. Dr. Elliott gave a very interesting account of the way in which they were made and fitted.

